# The Non-synonymous rs763780 Single-Nucleotide Polymorphism in IL17F Gene Is Associated With Susceptibility to Tuberculosis and Advanced Disease Severity in Argentina

**DOI:** 10.3389/fimmu.2019.02248

**Published:** 2019-09-20

**Authors:** Agustín Rolandelli, Joaquín Miguel Pellegrini, Rodrigo Emanuel Hernández Del Pino, Nancy Liliana Tateosian, Nicolás Oscar Amiano, María Paula Morelli, Florencia Andrea Castello, Nicolás Casco, Alberto Levi, Domingo Juan Palmero, Verónica Edith García

**Affiliations:** ^1^Department of Biological Chemistry, University of Buenos Aires (UBA), School of Sciences, Buenos Aires, Argentina; ^2^Institute of Biological Chemistry of Exact and Natural Sciences (IQUIBICEN), National Council of Science and Technology (CONICET), Buenos Aires, Argentina; ^3^Center of Investigation and Transference of National Northwest University of Buenos Aires (CITNOBA), The National Northwest University of Buenos Aires (UNNOBA)-CONICET, Buenos Aires, Argentina; ^4^Tisioneumonology Division, F. J. Muñiz Hospital, Buenos Aires, Argentina

**Keywords:** tuberculosis, Th17, IL17F, single-nucleotide polymorphism, rs763780, immunogenetics

## Abstract

Th17 lymphocytes, that produce IL17A, IL17F, and IL22, play a crucial role during the immune response against *Mycobacterium tuberculosis* (*Mtb*) infection. Whereas, the contribution of IL17A in immunity to tuberculosis is usually accepted, the role of IL17F has been scarcely studied so far. The aim of this work was to evaluate the existence of a potential association of the non-synonymous variant rs763780 SNP of the *IL17F* gene with human tuberculosis. Accordingly, by comparing healthy donors (HD) and tuberculosis patients (TB) populations we demonstrated an association between the C allele of the SNP and the susceptibility to tuberculosis disease in Argentina. Furthermore, we found that peripheral blood mononuclear cells (PBMCs) from individuals with a more effective immune response against *Mtb* secreted the highest levels of IL17F when stimulated with a lysate of *Mtb* (*Mtb*-Ag). Besides, we evidenced that *Mtb*-Ag-stimulated PBMCs from HD carrying the C variant of the SNP displayed the lowest IFNG secretion, proliferation index, and SLAM expression as compared to TT carriers. Moreover, *Mtb*-Ag-stimulated PBMCs from TB carrying the C allele produced the lowest levels of IFNG, the highest level of IL17A, and the minimum proliferation indexes as compared to TT TB, suggesting a relationship between the C allele and tuberculosis severity. In fact, TB carrying the C allele presented a more severe disease, with the highest bacilli burden in sputum. Together, our findings identify the *IL17F* rs763780 SNP as a biomarker of tuberculosis susceptibility and advanced disease severity in Argentina, suggesting that IL17F could be a critical cytokine in tuberculosis immunity.

## Introduction

*Mycobacterium tuberculosis* (*Mtb*) is the major cause of death by a microbiological agent, causing nearly 1.6 million of deaths per year. Moreover, *Mtb* infects almost 2 billon persons and causes nearly 10 million of new cases of tuberculosis annually (1). In Argentina, 11,503 cases of active tuberculosis and 910 deaths were reported in 2017 (1). Nevertheless, it is well-known that the majority of the subjects that were in contact with *Mtb* do not develop active disease, which might indicate that several factors, such as host genetic, and environmental causes, would affect the susceptibility to tuberculosis (2).

A successful immune response against *Mtb* depends on the activation of CD4^+^T lymphocytes, especially Th1 cells (3, 4). In fact, a reduced secretion of IFNG by T cells is a marker of the severity of the disease. Consequently, patients with tuberculosis (TB) displaying the most severe expression of the disease produce the lowest amounts of IFNG against the pathogen (5, 6). Moreover, we have reported that the *IFNG* rs1861494 single nucleotide polymorphism (SNP) would act as a biomarker of resistance to tuberculosis in the Argentinean population, since it affects the secretion of IFNG (7). However, other cytokines might be participating in the fight of the host against *Mtb* infection, given that only IFNG is not enough to completely eliminate the bacteria (8).

Several reports have proposed that the secretion of IL17A, IL17F, and IL22 by Th17 cells contribute to the immune response of the human host against *Mtb* (9). For example, IL17A is produced by CD4^+^ T lymphocytes in order to eliminate the primary infection, and to establish an effective memory response (10–14). Recently, we found that IL17A augments autophagy in *Mtb*-infected monocytes from individuals with strong immunity to the bacterium; but this cytokine was unable to increase autophagy levels in monocytes from TB with severe disease, at least in part, because of a defect in the MAPK1/3 signaling pathway (15). However, when the amounts of IL17A are too elevated they might be harmful for the host, leading to an excess of neutrophils' recruitment that might induce an exacerbated inflammation and tissue damage (11). We also reported that *Mtb*-Ag stimulated PBMCs from TB secreted minor levels of IFNG and greater amounts of IL17A as compared to healthy donors (HD) (16). Moreover, we demonstrated that CD4^+^IFNG^+^IL17A^+^cells from TB produce the highest levels of IL17A, in direct correlation with the severity of the disease (16). Finally, whereas we recently showed a positive association of the *IL17A* rs2275913 SNP with protection against active tuberculosis, we also demonstrated that *IL17A* rs2275913 SNP is related to advanced disease in the Argentinean population (17).

IL17F is another important cytokine produced by the Th17 subset. Both IL17A and IL17F are members of the IL17 cytokine family. In humans, their genes are located adjacent to one another in the chromosome 6p12, they share 55% of sequence homology and they have similar expression profiles (18, 19). IL17A and IL17F can form either homodimers or heterodimers, and their signaling is through a dimeric IL17RA and IL17RC receptor complex (20). Functionally, both IL17A and IL17F participate in the inflammation process by inducing the production of other cytokines and chemokines, and the expression of antimicrobial genes in fibroblasts, or endothelial cells (21). Strikingly, these two similar cytokines may also have different, or even opposite, functions in certain cases. For example, in the murine model of experimental autoimmune encephalomyelitis (EAE), only *il17a*^−/−^ mice show a significant reduction in the severity of the disease, indicating a role for IL17A but not for IL17F. In addition, IL17A promotes inflammation in an asthma murine model, with reduced infiltration of eosinophils in the airways of *il17a*^−/−^ mice. In contrast, *il17f* ^−/−^ animals show a greater infiltration of Th2 cytokines and eosinophils, suggesting a suppressive function of IL17F in asthma. It has also been demonstrated that IL17A is protective while IL17F is harmful in certain types of colitis (22, 23). Regarding the role of IL17A and IL17F in the immunity against infections, these cytokines seem to have redundant functions in the defense against *Staphylococcus aureus*, although both cytokines are necessary in the control of *Citrobacter rodentium* infection (22). Moreover, it has been shown that patients with hyper-IgE syndrome, who have a mutation in the *STAT3* gene that leads to a defective IL17A/F production, are highly susceptible to *Staphylococcus aureus, Streptococcus pneumonia*, and *Candida albicans* infection (24).

Until now, there is limited information regarding the role of IL17F during the human immune response against *Mtb*. Accordingly, it has been reported that IL17F is produced by whole blood stimulated with *Mtb*-Ag and by mucosal-associated invariant T cells (MAIT) (25, 26). Furthermore, human and mice deficient in *RORGT*, which show a significant decrease in the lymphocyte production of IL17A and IL17F, display disseminated infection by BCG and *Mtb* (27). In leprosy, IL17F plasma levels are inversely proportional to the bacteriological index. However, antigen-stimulated-PBMCs from leprosy patients with the most severe form of the disease produced the highest amounts of IL17F (28, 29). Overall, these findings suggest an important role for IL17F in the immunity against mycobacteria.

The *IL17F* rs763780 SNP, a non-synonymous variant that cause a His-to-Arg (H161R) substitution, is located in the third exon of the *IL17F* gene, and was demonstrated to cause a loss in the ability of IL17F to induce expression of certain cytokines and chemokines (30). Moreover, it has been reported that the H161R variant acts as a natural antagonist of the wild-type IL17F, because it can bind to its receptor but without triggering a signal, blocking the induction of IL8 expression (30, 31). Besides, several reports have shown the association of the *IL17F* rs763780 SNP with different inflammatory diseases, such as rheumatoid arthritis, inflammatory bowel disease, asthma, Graves' disease, ulcerative colitis, cancer, among others (32–39). Until now, the association between the rs763780 SNP and tuberculosis in the Argentinean population has not been explored.

Given that IL17F induces chemokines and antimicrobial peptides in the lung, and considering the cross regulation of Th1 and Th17 responses during mycobacterial infections, we hypothesized that IL17F participates in the human immune response against *Mtb*. Therefore, in the present study, we used an *in vitro* human model of primary cell cultures to assess the production of IL17F by *Mtb*-Ag-stimulated PBMCs from HD and TB. Moreover, we analyzed the association of the *IL17F* rs763780 SNP with tuberculosis disease in Argentina, evaluating the potential relationship of this polymorphism with immunological and clinical parameters of the severity of the disease.

## Materials and Methods

### Samples

Patients with active tuberculosis (TB) were diagnosed at the Hospital Muñiz (Buenos Aires, Argentina), based on clinical and radiological data, together with the identification of acid-fast bacilli in sputum and isolation of *Mtb* in culture. Patients recruited were individuals vaccinated with Bacille Calmette Guerin *Mycobacterium bovis* (BCG), negative for HIV, with no underlying diseases (cancer, diabetes, chronic obstructive pulmonary disease, immune-related diseases, and others). In addition, they had received <1 week of anti-tuberculosis therapy at the time the sample was obtained. TB were classified as High Responder (HR) patients (individuals displaying high proliferative responses, IFNG production, and SLAM expression in CD3^+^ cells against *Mtb*-Ag), and Low Responder (LR) patients (subjects that exhibit low proliferative responses, IFNG secretion, and percentages of SLAM^+^ CD3^+^ cells), as previously described (6). Healthy donors (HD) recruited were individuals vaccinated with BCG who lack history of tuberculosis, negative for HIV, and with no underlying diseases (cancer, diabetes, immune-related diseases, and others) or any pharmacological treatment at the time of recruitment. All HD were tested using the QuantiFERON-TB Gold In-Tube test (QFT-Qiagen, Hilden, Germany), and only QFT negative individuals were included in this group. Subjects with latent tuberculosis (positive QFT) were excluded from the study. All the individuals participating in this study were over 18 years old.

All participants provided written, informed consent for the collection of samples and subsequent analysis, in accordance with the Declaration of Helsinki. The protocols conducted in this work were approved by the Ethical Committee of the Dr. F. J. Muñiz Hospital.

### Antigen

*In vitro* stimulation of cells throughout the study was performed with a cell lysate from the virulent *M. tuberculosis* H37Rv strain, prepared by probe sonication (*Mtb*-Ag), and obtained through BEI Resources, NIAID, NIH: *Mycobacterium tuberculosis*, Strain H37Rv, Whole cell lysate, NR-14822 (Bethesda, MD, USA).

### DNA Extraction, SNP Primers Design, and Genotyping

Genomic DNA was extracted from whole blood samples using the Quick-gDNA™ Blood MiniPrep (ZymoReasearch, California, USA) according to the manufacturer's instructions. DNA purity and final concentrations were determined spectrophotometrically. Amplification refractory mutation system-polymerase chain reaction (ARMS-PCR) was used for the rs763780 SNP genotyping. The ARMS-PCR is based on allele specific amplification of desired fragment using primers corresponding to each allelic variant (40). Primer sequences were designed by the Beacon Designer 7.2 software (Premier Biosoft International, Ltd., CA, USA). The sequences of the primers used were: Allele T specific forward 5′ GGATATGCACCTCTTACTGCACTT 3′, Allele C specific forward 5′ GGATATGCACCTCTTACTGCACTC 3′, Common reverse 5′ CACCAAGGCTGCTCTGTTTCTT3′. As an internal control, Human Growth Hormone (*HGH*) gene primers (Forward 5′ gccttcccaaccattccctta 3′, Reverse 5′ TCACGGATTTCTGTTGTGTTTC 3′) were included in every PCR mix to verify successful amplification. The amplification was performed in a Multigene Gradient thermal cycler (LabNet International, NJ, USA). The conditions included initial denaturation (94°C for 5 min) following a 35 time cycles of denaturation at 94°C for 30 s, annealing at 60°C for 50 s, and extension at 72°C for 45 s each cycle; and final extension at 72°C for 5 min. The rs763780 genotypes were assessed from the presence/absence of PCR amplicon (106 bp), corresponding to the specific allele (T/C) on 1.5% agarose gel stained with SYBR Green. All genotypes of the rs763780 SNP were confirmed by direct sequencing of the amplified *IL17F* gene fragment by Sanger method (ABI 3130xl Genetic Analyzer, Applied Biosystems, USA), and a 100% concordance was obtained among the results obtained from ARMS-PCR and DNA sequencing (Supplementary Figure S1).

### Cell Preparation and Reagents

Peripheral blood mononuclear cells (PBMCs) were isolated by centrifugation over Ficoll-Hypaque (Amersham Biosciences, NJ, USA) and cultured (1 × 10^6^ cells/mL), with or without *Mtb*-Ag (10 μg/mL) with RPMI 1,640 medium (Gibco, MD, USA) supplemented with 1% L-glutamine, 1% penicillin/streptomycin, and 10% human serum (Sigma-Aldrich, MO, USA) during 5 days. Then, IL17F (RyD Systems), IL17A (eBioscience), and IFNG (BioLegend) secretion was measured in cell-free supernatants by ELISA, following the manufacturers' instructions.

### Flow Cytometry

PBMCs were stimulated with *Mtb*-Ag for 4 (IL17F detection) or 5 days (IL17A, IFNG, and SLAM detection), and incubated with monensin (1 μl/ml; Sigma-Aldrich, MO, USA) for the last 5 h of culture. Cells were then stained with specific fluorophore-marked antibodies against CD3 (FITC, UCHT1, BioLegend), CD4 (FITC, RPA-T4, BioLegend), and SLAM (PE, A12, BD Pharmingen). Intracellular staining was performed to determine IL17F (eFluor660, SHLR17, eBioscience), IL17A (PE-Cy7, eBio64DEC17, eBioscience), and IFNG (APC, 4S.B3, eBioscience) expression. For intracellular cytokine staining, permeabilization buffer containing 0.5% saponin (Sigma-Aldrich, MO, USA), and 10% fetal bovine serum (Gibco, MD, USA) in PBS was used. Negative control samples were incubated with irrelevant isotype-matched mAb in parallel with experimental samples, which were analyzed on a FACSAria II flow cytometer (BD Biosciences). To identify the different cytokines produced by T lymphocytes, the gating strategy comprised an initial determination of the blast lymphocytes area according to their forward and side scatter properties, followed by the discrimination of CD4^+^cells based on FITC fluorescence. Finally, IL17F, IFNG, or IL17A^+^ cells were identified by staining with specific antibodies bound to the different fluorophores (Supplementary Figure S2).

### Proliferation Index

PBMC were stimulated with *Mtb*-Ag for 5 days and cells were pulsed with [^3^H]TdR (1 μCi/well) and harvested 16 h later. [^3^H]TdR incorporation was measured in a liquid scintillation counter. Proliferation index for each individual was calculated as cpm after *Mtb*-Ag stimulation/cpm after culturing with medium.

### Statistical Analysis

The genotype and allele frequencies were obtained by direct counting. Hardy-Weinberg (HW) equilibrium was tested between cases and controls separately (Chi-Square goodness-of-fit test). Comparisons of the distributions of the allele and genotype frequencies between case and control were performed using the Chi-Square test with Yates correction or Fisher exact test. The level of association between the rs763780 genotypes and the case/control condition was estimated as an odds ratio (OR) with a 95% confidence interval (C.I.), calculated by logistic regression after adjusting for confounding variables (age/ethnicity/sex) (41). An *a priori* statistical analysis to determine the final sample size was performed with an initial population of 100 HD and 100 TB. The sample size estimation to get a test power of 0.8, using the initial population HD minor allele frequency (MAF) of 0.035 and the TB MAF of 0.095, was of at least 164 individuals in each population. The quantitative data were expressed as mean ± standard error of the mean (SEM), and the Mann–Whitney *U*-test for unpaired and non-parametric samples or the Wilcoxon W rank sum test for paired and non-parametric samples were used to analyze differences between groups. For categorical variables, the Chi-Square test for homogeneity was performed to compare proportions of subjects between groups and the Chi-Square goodness-of-fit test was used to evaluate deviations from Hardy-Weinberg equilibrium. All statistical analysis were performed using GraphPad Prism v6.0 (GraphPad Software, CA, USA) or the R software (42). *p* <0.05 were considered statistically significant.

## Results

### Demographic Characteristics of the Population Studied

Demographic characteristics of HD and TB populations are shown in Table 1. Both populations are comparable in terms of ethnicity (*p* > 0.05) and age (*p* > 0.05), but displayed different sex proportions (*p* < 0.001). Taking into consideration that the differences in the proportions of sexes could affect the results of genetic association studies, both populations under study were stratified by sex, and the genotypic frequencies distribution of the *IL17F* rs763780 SNP was calculated (Table 2). Given that we did not find differences, we determined that the disparities between the percentages of subjects from each sex would not impact in the genotype distribution investigated in each population.

**Table 1:** Demographic characteristics of healthy donor (HD) and tuberculosis patient (TB) populations.

		**HD**	**TB**	***p*-Value**
*n*		201	200	
Age (Years)		33.6 ± 1.0	33.0 ± 1.0	0.103*
Ethnicity	Caucasian	68.45%	64.07%	0.343^X^
	American Indian	31.55%	35.93%	
Sex	Male	38.31%	73.00%	** <0.001**^X^
	Female	61.69%	27.00%	

Categorical variables are expressed in percentages. Age value is expressed as Mean ± Standard Error of the Mean (SEM). Statistical differences were calculated using the Mann-Whitney U-test for unpaired samples (*) and the Chi-Square (χ^2^) test for categorical variables (X). n: number of individuals.

*Bold numbers indicate statistically significant differences between groups, with a p-value <0.05*.

**Table 2:** Genotypic frequencies of the *IL17F* rs763780 SNP in HD and TB populations stratified by sex.

**rs763780 SNP genotypes**		**HD (*****N*** **=** **201)**		**TB (*****N*** **=** **200)**	
		**TT**	**TC/CC**	**TT**	**TC/CC**
Sex	Male	65	12	102	44
	Female	105	19	36	18
*p*-Value		0.991		0.674	

*p-Values were calculated by the Chi-Square (χ^2^) test for categorical variables. HD, healthy donors; TB, tuberculosis patients*.

### The C Variant of the IL17F rs763780 SNP Is Associated With Susceptibility to Tuberculosis Disease

Genotyping of the rs763780 SNP in the populations of HD and TB was performed by the ARMS-PCR technique as described in Methods (Supplementary Figure S1). Remarkably, no individual with the CC genotype was found in the HD population, whereas three subjects carrying the CC genotype (out of 200) were detected in the population of TB. For that reason, all the statistical analyzes were performed in C carriers (TC+CC genotypes), or non-C carriers (TT genotype).

**Figure 1:**
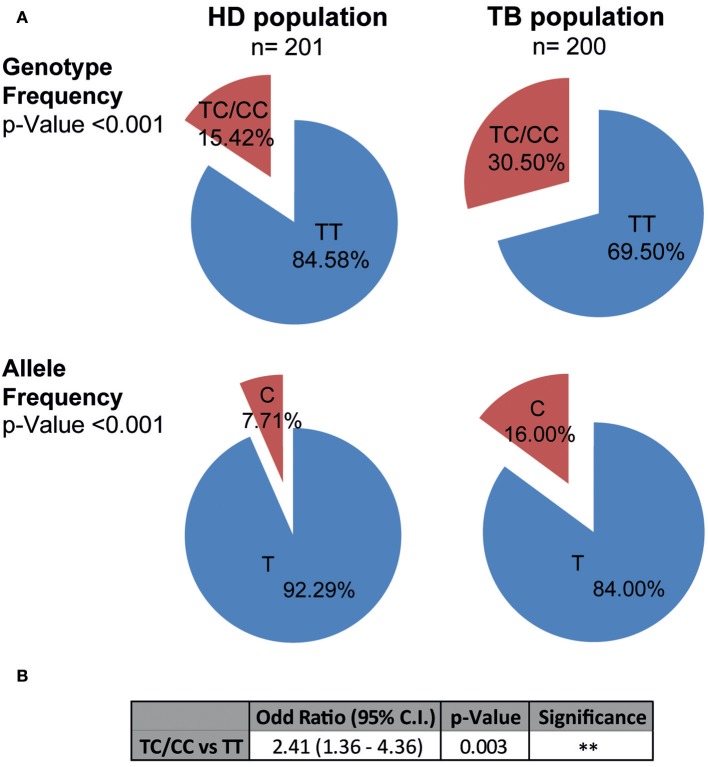
Genotypic and allelic frequencies of the *IL17F* rs763780 SNP in HD and TB populations in Argentina. **(A)** Pie chart representing the genotypic and allelic distribution of the rs763780 SNP in healthy donor (HD, *n* = 201) and tuberculosis patient (TB, *n* = 200) populations. The number of individuals of each population and the frequencies are detailed. Statistical differences were calculated using the Chi-Square (χ^2^) test of homogeneity. Both populations are in Hardy-Weinberg equilibrium. **(B)** Odds ratio was calculated by logistic regression after adjusting for confounding variables (age/ethnicity/sex), in order to quantify the association between tuberculosis and the different genotypes.

Figure 1A shows the genotypic and allelic frequencies distribution observed in HD and TB populations. Notably, both populations were in Hardy-Weinberg (HW) equilibrium. Chi-Square test of homogeneity determined that HD and TB populations were significantly different regarding the genotypic and allelic frequencies (*p* < 0.001, for genotypes and for allele distribution). In fact, we detected a higher proportion of individuals carrying the C variant of the SNP in TB patients as compared to HD (TC+CC genotype: TB 30.50% vs. HD 15.42%, *p* < 0.001). Moreover, we found a significantly higher proportion of the C allele in the TB population (C allele: TB 16.00% vs. HD 7.71%, *p* < 0.001). Odds ratios were calculated to estimate the association level between the TC+CC genotype (C carriers) and tuberculosis disease (Figure 1B) (41). When comparing individuals carrying the C allele against non-C carriers, we observed an odd ratio of 2.41 (95% C.I. = 1.36–4.36; *p* < 0.01), indicating an association between C carriers and tuberculosis susceptibility in Argentina. Thus, these data demonstrate a relationship between the C allele of the rs763780 SNP and a higher frequency of individuals suffering from tuberculosis, suggesting a role for IL17F in the development of the disease.

### IL17F Production Is Higher in Individuals With a More Effective Immune Response Against *Mtb*

**Figure 2:**
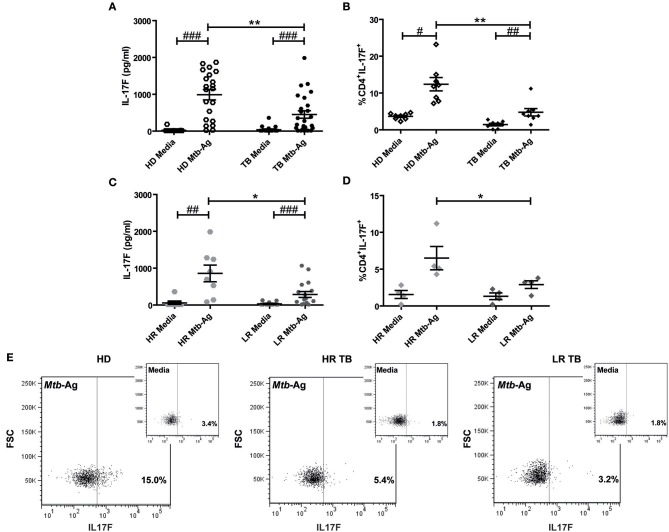
Production of IL17F by *Mtb*-Ag stimulated PBMCs from HD and TB. **(A)** Peripheral Blood Mononuclear Cells from healthy donors (HD, *n* = 21), and tuberculosis patients (TB, *n* = 25) were cultured for 5 days either alone or with a lysate of *Mtb* (*Mtb*-Ag). Afterwards, IL17F production was determined in culture supernatants by ELISA. **(B)** PBMCs from HD (*n* = 8) and TB (*n* = 8) were cultured for 4 days either alone or with *Mtb*-Ag, and IL17F production were determined by Flow Cytometry. **(C,D)** TB were classified as High and Low Responder (HR and LR, respectively) individuals according to their immune response to *Mtb*-Ag. IL17F production by stimulated PBMCs was determined by **(C)** ELISA and **(D)** Flow Cytometry. **(E)** Representative dot plots for each group (HD, HR TB, and LR TB) are shown. IL17F secretion was determined by first gating on lymphocytes by light scatter, and then by gating on CD4^+^ T cells. Lines represent the Mean ± Standard Error of the Mean (SEM). Statistical differences were calculated using the nonparametric Mann-Whitney *U*-test for unpaired samples (*p < 0.05 and **p < 0.01), and the Wilcoxon W rank sum test for paired samples (^#^*p* < 0.05, ^##^*p* < 0.01, and ^###^*p* < 0.001).

To further investigate the potential role of IL17F in tuberculosis, we determined the production of this cytokine by PBMCs from HD and TB. In order to optimize the detection of IL17F, we initially performed a kinetic analysis. Thus, PBMCs from HD were stimulated with *Mtb*-Ag and the production of this cytokine was evaluated at 16, 48, and 120 h of culture. Our data showed maximum IL17F production after 120 h (5 days) of Ag stimulation (Supplementary Figure S3). Next, we analyzed the ability of PBMCs from TB to secrete IL17F and compared it to HD. Our findings showed that *Mtb*-Ag stimulation induced significant IL17F production by PBMCs from TB (*p* < 0.001; Figure 2A). Strikingly, and in sharp contrast with our reports studying IL17A production, we detected higher levels of IL17F in *Mtb*-Ag stimulated PBMCs from HD than in TB (*p* < 0.01; Figure 2A), suggesting that these cytokines may play different roles during tuberculosis disease (16, 17). Further validation of the above data was obtained by flow cytometry analysis. Accordingly, we were able to determine that CD4^+^ T cells produced IL17F, observing the maximum differences in the percentages of IL17F producing cells between HD and TB after 4 days of *Mtb*-Ag stimulation (Supplementary Figure S4). Furthermore, our data indicated that HD displayed significantly higher numbers of CD4^+^IL17F^+^ lymphocytes as compared to TB (*p* < 0.01, Figure 2B).

By using immunological parameters, we previously differentiated two populations of TB in Argentina, High, and Low Responders (HR and LR, respectively), where LR subjects suffer the most advanced disease (6, 16, 43). In order to further investigate whether subjects with a more effective immune response against *Mtb* displayed higher IL17F production, we analyzed the secretion of this cytokine by HR and LR TB. As shown in Figure 2C, *Mtb*-Ag stimulated PBMCs from HR secreted higher amounts of IL17F as compared to PBMCs from LR (*p* < 0.05). It is important to note, the levels of IL17F detected in HR were similar to the levels measured in HD (Figure 2A). In line with the ELISA results, we found that the greatest percentages of CD4^+^ T cells producing IL17F were observed in *Mtb*-Ag stimulated PBMCs from HR (*p* < 0.05; Figures 2D,E). Overall, these results indicate that individuals that mount a more effective immune response against the bacterium secret the highest amounts of IL17F, and suggest that IL17F might have a protective role in human tuberculosis.

### PBMCs From Individuals Carrying the C Variant of the IL17F rs763780 SNP Displayed a Reduced Immunological Protection Against *Mtb*

**Figure 3:**
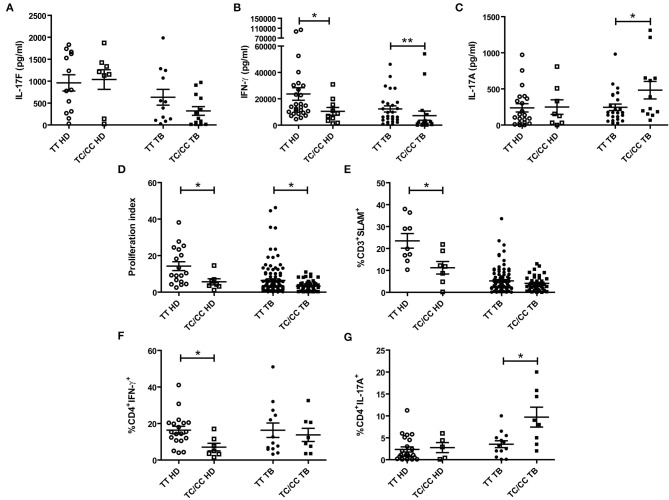
Association between the IL17F rs763780 SNP genotypic variants and immunological parameters in tuberculosis. Peripheral Blood Mononuclear Cells from healthy donors (HD) and tuberculosis patients (TB) carrying the different genotypes of the rs763780 SNP (TT and TC/CC) were stimulated for 5 days with a lysate of *Mtb* (*Mtb*-Ag). Afterwards, IL17F, IFNG, and IL17A secretion were determined by ELISA (**A–C**, respectively). The proliferation index (cpm from stimulated cells/cpm from un-stimulated cells) was evaluated by [^3^H] thymidine incorporation **(D)**; and SLAM expression in CD3^+^ T cells was determinate by Flow Cytometry **(E)**. Additionally, CD4^+^ T cells production of IFNG **(F)** and IL17A **(G)** was determined by Flow Cytometry. Lines represent the Mean ± Standard Error of the Mean of the increase in response to *Mtb*-Ag stimulation. Statistical differences were calculated using the non-parametric Mann-Whitney *U*-test for unpaired samples (*p < 0.05 and **p < 0.01).

To get additional evidence on the role of IL17F in tuberculosis, we wondered whether individuals carrying the C variant of the rs763780 SNP would display a weaker immune response against *Mtb*. Thus, PBMCs from HD and TB, discriminated in C carriers, or non-C carriers, were stimulated with *Mtb*-Ag, and we evaluated IL17F, IFNG, and IL17A production, proliferation index and SLAM expression (Figure 3). We found no differences between IL17F levels produced by HD and TB carrying the different SNP variants (Figure 3A). However, considering that the C allele codifies for a non-synonymous protein, individuals carrying the C allele could mount a different immune response as compared to TT individuals. In fact, *Mtb*-Ag stimulated PBMCs from HD carrying the C allele showed significant lower production of IFNG and lower percentages of CD4^+^ IFNG^+^ T cells as compared to TT HD (*p* < 0.05, Figures 3B,F, Supplementary Figure S5). In the same way, *Mtb*-Ag stimulated PBMCs from TB carrying the C allele showed significant lower secretion of IFNG as compared to TT TB (*p* < 0.01, Figure 3B). Given the crucial role of IFNG in the protection against *Mtb* infection (3–6), these results are in line with the genetic association found between the C carriers of the rs763780 SNP and the susceptibility to tuberculosis.

We have previously demonstrated that IL17A production correlates with disease severity in TB (16, 17). We found no differences in the secretion of IL17A by HD carrying the different variants of the *IL17F* rs763780 SNP (Figures 3C,G). In contrast, we detected higher IL17A secretion and higher percentages of CD4^+^ IL17A^+^ T cells in TB carrying the C allele, as compared to TT TB (*p* < 0.05, Figures 3C,G, Supplementary Figure S5), suggesting a relationship between this allele and the severity of the disease. Furthermore, we observed that both the levels of SLAM and the proliferation index were lower in HD carrying the C allele (*p* < 0.05, Figures 3D,E), in line with our data showing an association of this allele and the susceptibility to tuberculosis. Besides, TC/CC TB displayed a significantly lower proliferation index as compared to TT TB (*p* < 0.05, Figure 3D), in close relationship with higher disease severity in TB carrying the C allele. Overall, we showed that individuals carrying the C allele of the *IL17F* rs763780 SNP displayed a weaker immune response against *Mtb*, supporting a potential role for IL17F in tuberculosis.

### The C Variant of the IL17F rs763780 SNP Is Highly Represented in Patients With More Severe Tuberculosis

**Figure 4:**
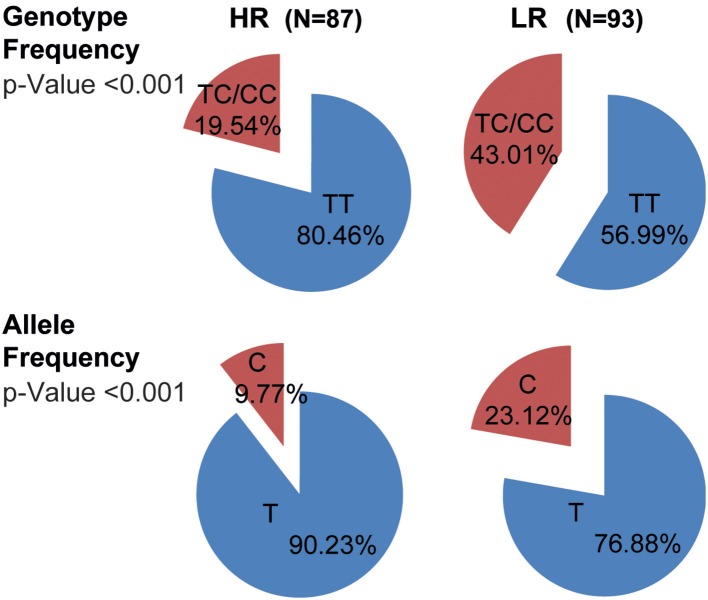
Genotypic and allelic frequencies of the *IL17F* rs763780 SNP in HR and LR subpopulations of TB. Pie chart representing the genotypic and allelic distribution of the rs763780 SNP in tuberculosis patients (TB, *n* = 180) classified as High and Low Responder (HR and LR, respectively) individuals according to their immune response to *Mtb-*Ag. The number of individuals in each group and the frequencies are detailed. Statistical differences were calculated using the Chi-Square (χ^2^) test of homogeneity.

Considering that PBMCs from TB carrying the C allele of the rs763780 SNP secreted the lowest levels of IFNG and the highest IL17A amounts against *Mtb*-Ag, and displayed the lowest proliferation index, we next investigated whether this polymorphism was related with the severity of the disease. Then, we determined the genotypic and allelic distribution of the rs763780 SNP in TB classified as HR and LR (6) (Figure 4). We found that patients carrying the C variant were present in higher frequency in the LR subpopulation of TB as compared to HR (TC+CC genotype: LR 43.01% vs. HR 19.54%; *p* < 0.001). Also, we observed that the C allele was present in a higher proportion in the subpopulation of patients with the most severe tuberculosis (C allele: LR 23.12 vs. HR 9.77%; *p* < 0.001). Thereby, our present findings demonstrate by genetic analysis and immunological studies that TB carrying the C allele of the *IL17F* rs763780 SNP display the weakest immune responses against *Mtb*-Ag, and therefore they would be severely affected by the disease.

### TB Carrying the C Allele of the IL17F rs763780 SNP Displayed the Highest Bacilli Burden in Sputum Smear

Previously we showed that immunological attributes matched clinical parameters studied in TB in Argentina (6, 16, 43). Thus, we next decided to analyze whether there was a relationship between clinical parameters commonly studied in tuberculosis and the genetic variants of the rs763780 SNP. Table 3 shows no significant differences in leukocyte, lymphocyte, monocyte, or neutrophil numbers in peripheral blood of TB carrying the different genotypes of the rs763780 SNP. Furthermore, we did not find differences either in the radiological pulmonary lesions or in the time of disease progression. However, when we analyzed the genotype frequencies of the IL17F SNP according to the bacillary load categories, we found significant differences among TC/CC and TT carriers (BAAR^−^,^+^, ^++^, and ^+++^; *p* < 0.05, Table 3). In fact, when we examined the genotype frequencies comparing the BAAR^−^, BAAR^+^, BAAR^++^, and BAAR^+++^ groups individually, significant differences were detected between BAAR^+^ and BAAR^+++^ (*p* < 0.01, significant after Bonferroni correction, Table 3). Interestingly, TB carrying the TT genotype presented the highest proportion of patients with low bacillary load (56.76% BAAR^+^) and the lowest percentages of individuals with the highest bacilli load (20.72% BAAR^+++^). In contrast, 40% of patients carrying the C allele displayed the most elevated bacillary load (BAAR^+++^), and a lower percentage of BAAR^+^ (28.89%). In fact, when we performed a cumulative comparison of the genotype frequencies between patients with the lowest bacillary load (BAAR^−^ and ^+^) and patients with the highest bacillary load (BAAR^++^ and ^+++^), significant differences were found (*p* < 0.01, Table 3). The majority of TB carrying the C allele showed the highest bacillary load (BAAR^++^ and BAAR^+++^: 62.7%), whereas a significant lower fraction of patients carrying the TT phenotype displayed BAAR^++^ or BAAR^+++^ bacillary load (39.34%). In contrast, the majority of TT TB presented the lowest bacillary load (BAAR^−^ or BAAR^+^: 60.66%), while the minority of TB carrying the C allele displayed low bacillary load (37.25%). Our findings would indicate that TB that carry the C allele of the IL17F rs763780 SNP display weak cell-mediated immunity against *Mtb* in association with higher bacilli load. Thus, taken together, the present results suggest that the TC/CC genotype of *IL17F* SNP might be related to the severity of the disease, highlighting a role of this cytokine during active tuberculosis.

**Table 3:** Association between the *IL17F* rs763780 SNP genotypic variants and clinical parameters of tuberculosis severity.

**TB population**	**rs763780 SNP Genotypes**		***p*-Value**
	**TT**	**TC/CC**	
**Hematologic studies (*****n*** **=** **123)**			
Leucocytes (cells/mL)	10 105 (± 449.6)	9 113 (± 540.4)	0.400*
Lymphocytes (cells/mL)	1 576 (± 71.70)	1 394 (± 115.3)	0.161*
Monocytes (cells/mL)	874.7 (± 46.81)	716.2 (±76.09)	0.092*
Neutrophils (cells/mL)	6810 (± 435.8)	7 423 (± 1 100)	0.759*
**AFB in sputum smear (*****n*** **=** **173)**			
BAAR^−^	9.02% (*n* = 11)	11.76% (*n* = 6)	**0.013**^X#^
BAAR^+^	51.64% (*n* = 63)	25.49% (*n* = 13)	
BAAR^++^	20.49% (*n* = 25)	27.45% (*n* = 14)	
BAAR^+++^	18.85% (*n* = 23)	35.30% (*n* = 18)	
BAAR^−^ or BAAR^+^ (Low bacillary 1^+^and negative)	60.66% (*n* = 74)	37.25% (*n* = 19)	**0.007**^X^
BAAR^++^ or BAAR^+++^ (High bacillary 2^+^and 3^+^)	39.34% (*n* = 48)	62.75% (*n* = 32)	
**Radiological lesions (*****n*** **=** **163)**			
Mild or Moderate	44.07% (*n* = 52)	40.00% (*n* = 18)	0.724^X^
Severe	55.93% (*n* = 66)	60.00% (*n* = 27)	
**Months of disease progression (*****n*** **=** **118)**	2.89 (± 0.28)	2.66 (±0.29)	0.848*

Hematologic studies representing the leukocyte, lymphocyte and monocyte counts in peripheral blood are shown. Acid-Fast Bacilli (AFB) in sputum smear represent: BAAR^−^, 0 bacilli count; BAAR^+^, 1-9 bacilli/ 100 fields; BAAR^++^, 1–9 bacilli/ 10 fields; BAAR^+++^, 1–9 bacilli/ field. Radiological lesions: mild corresponds to patients with a single lobe involved and without visible cavities; moderate relates to patients presenting unilateral involvement of two or more lobes with cavities, if present, reaching a diameter no >4 cm; severe corresponds to bilateral disease with massive affectation and multiple cavities. Clinical symptoms analyzed in TB before hospital admission to establish the time (months) of disease evolution were: weight loss, night sweats, symptoms of sickness or weakness, persistent fever, presence of cough, history of shortness of breath, and hemoptysis. Continuous data are expressed as Mean (± SEM), and categorical data are expressed as percentages of genotype (n: number of individuals). Statistical differences were calculated using the Mann-Whitney U-test for unpaired samples (*) and the Chi-Square (χ^2^) test for categorical variables (X).

# *Individuals comparisons between AFB categories: p = 0.1058 for BAAR^−^ vs. BAAR^+^; p = 1.0000 for BAAR^−^ vs. BAAR^++^; p = 0.5750 for BAAR^−^ vs. BAAR^+++^; p = 0.0357 for BAAR^+^ vs. BAAR^++^; p = 0.0038 for BAAR^+^ vs. BAAR^+++^; p = 0.5009 for BAAR^++^ vs. BAAR^+++^. Bonferroni correction for multiple comparisons: α = 0.05/6 = 0.008. Bold numbers indicate statistically significant differences between groups, with a p-value <0.05*.

## Discussion

It is widely accepted that Th17 cells, that produce IL17A and IL17F, contribute to the adaptive immunity to *Mtb* (9). Nevertheless, while the contribution of IL17A to mycobacteria immunity is usually accepted, the role of IL17F in tuberculosis disease has been poorly studied to date. Therefore, in this study we assessed the production of IL17F by *Mtb*-Ag-stimulated cells from HD and TB. Moreover, we evaluated the relevance of the *IL17F* rs763780 SNP during human infection caused by *Mtb*.

We determined the production of IL17F using an *in vitro* model of human primary cell cultures stimulated by *Mtb*-Ag. Our findings demonstrated that individuals that mount an effective immune response against *Mtb* secreted the highest levels of IL17F and showed the greatest percentages of CD4^+^IL17F^+^T cells. Accordingly, whole blood stimulation with specific *Mtb* antigens showed an increase in the production of IL17F in individuals with latent tuberculosis infection (LTBI) as compared to TB (25). Furthermore, uncontrolled infections with BCG or *Mtb* were reported in individuals with defects in IL17A and IL17F production (27). Besides, it has been also reported that MAIT cells from TB produced higher levels of IL17F at the site of infection than in the periphery (26).Thus, our present findings, together with previous reports, suggest a protective role for IL17F during the immune response of the host against mycobacteria.

Unlike IL17A, which is mainly produced by T cells and innate lymphoid cells, recent study shows that IL17F is secreted by almost every cell populations, including myeloid-derived cells and even organic epithelial or endothelial cells (44, 45). Therefore, here we analyzed the secretion of IL17F by other PBMCs populations besides CD4^+^. However, our results showed very low percentages of CD14^+^ and CD8^+^ IL17F^+^ secreting cells, with no significant differences between media and *Mtb*-Ag condition (data not shown). Then, we hypothesize that the contribution of IL17F secretion by PBMCs other than CD4^+^ is minimal. Nevertheless, we cannot exclude the potential contribution of IL17F production by other cell populations at the site of the infection that were not analyzed in this study, so further studies would be required to confirm our hypothesis.

The rs763780 SNP is located in the coding region (position +7488) of the *IL17F* gene. It consists of a T → C substitution, which generates a change in the amino acid sequence of IL17F protein: from Histidine to Arginine in position 161 (H161R). *In vitro* functional experiments demonstrated that the H161R variant of IL17F lacks the ability to activate the mitogen-activated protein kinase pathway, cytokine production, and chemokine secretion in bronchial epithelial cells (30). Furthermore, the H161R variant blocked the induction of the expression of IL8 by wild-type IL17F, acting as a natural antagonist of the cytokine (30, 31). On the other hand, Puel et al. also performed *in vitro* experiments with wild-type IL17F and the H161R variant (46). Nevertheless, they stimulated a murine lung epithelial cells line (MLE-12) and found that IL17F H161R variant induced the secretion of keratinocyte-derived chemokine (an homologous of human IL8) (46). Then, we believed that these discrepancies could be related to differences in the experimental design (human vs. murine cells). Besides, although human and murine IL17F sequences (and the IL17RA/C sequences) share some degree of similarity, they are not identical. These sequence differences could explain why the functionality of the IL17F H161R variant differs between human and mouse.

Several reports showed the association of this SNP with different diseases, mostly inflammatory, such as rheumatoid arthritis, inflammatory bowel disease, asthma, Graves' disease, ulcerative colitis and cancer, among others (32–39). In this work, we analyzed the association of the C variant of the rs763780 SNP with susceptibility to tuberculosis. Remarkably, more than 85% of individuals from the HD population were subjects exposed to *Mtb* but not infected with the pathogen (negative QFT). These individuals were recruited from families who had at least one patient living in the same household, having high probability of repeated exposure, making this population an appropriate control for the case-control study (47) and emphasizing that these individuals would be efficiently resistant to *Mtb* infection. Importantly, only three TB expressing the *IL17F* non-synonymous variant (CC genotype) were found, whereas no HD carrying the CC genotype were detected. These findings would suggest a role for IL17F in the development of the disease.

Ethnicity, sex and age differences between populations could cause unauthentic relationships in genetic association analyses, acting as confounding variables. In this work, we observed that both populations under study (HD and TB) were similar regarding age and ethnic composition. Notably, these ethnic proportions were comparable to the ethnic composition of the Argentinean population (48, 49). Although the HD and TB populations evaluated presented different sex proportions, we did not find discrepancies in the genotypic frequencies distribution of the *IL17F* rs763780 SNP in HD and TB stratified by sex. Additionally, the genotypes distribution in the HD and TB populations showed no deviation from HW equilibrium, indicating that there is no population substructure. Furthermore, the logistic regression used to evaluate the association between the *IL17F* rs763780 SNP and tuberculosis disease was adjusted for age, ethnicity and sex. In conclusion, we believe that it would be improbable that the mentioned variables acted as confounding variables in our genetic association analyses.

Even though other authors studied the relationship between the rs763780 SNP and tuberculosis, variable results were reported (50–55). We think that these inconsistencies might be associated with ethnic variances, differences in the selection of the control group and lack of HW equilibrium in the population analyzed, among other causes. In contrast to our present findings, studies conducted on Indian and Croatian populations found no association between this polymorphism and tuberculosis (54, 55). Nevertheless, our results are in agreement with studies performed in Chinese and Iranian populations, where an association between the SNP and the failure of anti-TB treatment was also demonstrated, supporting the statement that subjects with the C allele of the *IL17F* rs763780 SNP display a higher susceptibility to tuberculosis disease (50–53).

No differences were found between IL17F secretion by PBMCs from HD or TB carrying the different rs763780 SNP variants, as expected for a SNP located in the coding region of the gene. However, we detected that HD or TB carrying the non-synonymous variant of the *IL17F* gene displayed the lowest IFNG secretion. These results are in line with the genetic association discovered between the C variant and the susceptibility to tuberculosis, and suggest that the IL17F signaling pathway could modify *IFNG* expression during human tuberculosis. Actually, humans with altered production of IL17A, IL17F, and IL22, in association with insufficient IFNG production, displayed a defective control of *Mtb* (27). The transcription factor cAMP response element binding protein (CREB) was reported to act as a downstream signaling molecule for IL17F (56). Interestingly, Samten et al. showed that CREB increased *Mtb*-Ag-stimulated IFNG secretion by binding to the *IFNG* proximal promoter, providing a link between the two cytokines (57).

Our results indicate that IL17A and IL17F may have different roles in the context of tuberculosis. In fact, we previously described that IL17A levels were higher in TB and correlated with the severity of the disease whereas, in this work, we detected the highest amount of IL17F in *Mtb*-Ag-stimulated PBMCs from HD and HR TB (16). It is important to note that all the reagents that were used in this study detect IL17F/F or IL17A/A homodimers. It was recently reported that the IL17A/F heterodimer could form two topologically-distinct heterotrimeric complexes, with potentially different signaling properties (58). Thus, not only IL17A and IL17F homodimers but also IL17A/F heterodimers could participate in the immune response against *Mtb*.

We observed the maximum proportion of patients carrying the C variant in the LR subpopulation, individuals that display the most severe tuberculosis. We have previously reported that HR patients had significantly higher percentages of total lymphocytes and exhibited higher PPD diameters compared to LR patients; and that LR individuals had severe pulmonary lesions and a striking loss of weight compared to HR subjects (6, 16, 43). Surprisingly, by evaluating clinical parameters usually studied in tuberculosis, we did not find significant differences in the radiological pulmonary lesions of the patients carrying the different genotypes of the rs763780 SNP. Furthermore, we found similar numbers of leukocyte, lymphocyte, monocyte, and neutrophil counts in peripheral blood of TB. It is well-established that Th17 cytokines are linked to neutrophil influx at the site of infection (11, 59, 60). Thus, we believe that, to elucidate if TB carrying the C allele showed increased neutrophil recruitment to the site of infection, studies in bronchoalveolar lavages would be required. On the other hand, we found that most of the TB carrying the non-synonymous variant of the *IL17F* gene displayed the highest levels of IL17A and presented the highest bacilli burden in sputum, in contrast to most of the TT TB. In line with these results, we and other authors have reported that IL17A directly correlates with high antigen load (17, 61). Moreover, it was evidenced in leprosy that the bacteriological index was inversely correlated with the IL17F levels found in plasma (28). These findings indicate that TB carrying the C allele of the *IL17F* rs763780 SNP exhibit a weak cell-mediated immunity against *Mtb*-Ag, in direct association with elevated bacilli loads, which demonstrate a higher severity of the disease.

We found an association between the *IL17F* rs763780 SNP and different immunological and clinical parameters related to higher susceptibility to tuberculosis and disease severity, emphasizing the relationship between the SNP and the active form of the infection. Although we could not rule out the possibility of a non-causal association, like another functional polymorphism in strong linkage disequilibrium with the rs763780 SNP, the fact that the polymorphism under study is non-synonymic and has been reported to act as a natural antagonist of the wild-type IL17F (30, 31), makes it an excellent candidate for a direct or causal association (62). Previously, we described that the cytokine milieu plays a main role in the differentiation of CD4^+^Th effectors cells (16, 63). In fact, for example, we demonstrated that the addition of IL17A diminished the levels of IFNG (63). One of our hypotheses associated to a direct role of IL17F in TB, implies that different amounts of this cytokine could influence the levels of IL17A and IFNG secreted by HD and TB, affecting the onset of the infection. In addition, another of our theories establishes that IL17F could have an effect on several immunological parameters, based on our previous reports showing that the amounts of cytokines produced during *Mtb* infection can differentially modulate the expression of co-stimulatory molecules (such as SLAM), which in turn might regulate the responsiveness of T cell to the pathogen (63–65). The factors mentioned above, alone or in combination, may contribute directly or indirectly to the genetic association found between the C allele of the *IL17F* rs763780 SNP and the susceptibility to tuberculosis.

Overall, we demonstrated that IL17F secreted by PBMCs in the context of tuberculosis is produced in higher proportions in individuals with an effective immune response against *Mtb*. Also, by comparing the HD and the TB populations, we found genetic evidences of an association between the *IL17F* rs763780 C allele, and susceptibility to tuberculosis. These findings were further supported by *in vitro* analyses. Additionally, we found genetic evidences of a direct relationship between the C allele and the severity to tuberculosis, data sustained by immunological and clinical parameters. Taken together, our results identify the *IL17F* rs763780 SNP as a biomarker of tuberculosis susceptibility and disease severity in Argentina, and suggest that IL17F could play a key role in the immune response of the human host to *Mtb* infection.

## Data Availability Statement

All datasets generated for this study are included in the manuscript/Supplementary Files.

## Ethics Statement

All participants provided written, informed consent for the collection of samples and subsequent analysis, in accordance with the Declaration of Helsinki. The protocols conducted in this work were approved by the Ethical Committee of the Dr. F. J. Muñiz Hospital.

## Author Contributions

VG and AR designed the study. NC, AL, and DP were in charge of patient recruitment, diagnosis of active tuberculosis, and sample collection. AR, JP, RH, NT, NA, MM, and FC were responsible for processing samples and performing ELISA and flow cytometry analysis. AR was in charge of DNA extraction and genotyping. AR, JP, RH, and VG performed the data management and analysis. AR and VG wrote the manuscript. All authors contributed to data gathering and interpretation and to revision of the report.

### Conflict of Interest

The authors declare that the research was conducted in the absence of any commercial or financial relationships that could be construed as a potential conflict of interest.
