# Supplementary material for: The Non-synonymous rs763780 Single-Nucleotide Polymorphism in IL17F Gene Is Associated With Susceptibility to Tuberculosis and Advanced Disease Severity in Argentina
Source: Front Immunol. 2019 Sep 20;10:2248. doi: 10.3389/fimmu.2019.02248 (PMC6764169; doi:10.3389/fimmu.2019.02248)
Supplement: Supplementary file 1 [file Data_Sheet_1.PDF]

Supplementary Material

The Non-Synonymous rs763780 Single-Nucleotide Polymorphism in IL17F Gene Is Associated with Susceptibility to Tuberculosis and Advanced Disease Severity in Argentina.

Agustín Rolandelli[1,2], Joaquín Miguel Pellegrini[1,2], Rodrigo Emanuel Hernández Del Pino[3], Nancy Liliana Tateosian[1,2], Nicolás Oscar Amiano[1,2], María Paula Morelli[1,2], Florencia Andrea Castello[1,2], Nicolás Casco[4], Alberto Levi[4], Domingo Juan Palmero[4], Verónica Edith García*[1,2].

[1]Department of Biological Chemistry, University of Buenos Aires (UBA), School of Natural Sciences, Buenos Aires, Argentina.

[2]Institute of Biological Chemistry of Exact and Natural Sciences (IQUIBICEN), National Council of Science and Technology (CONICET), Buenos Aires, Argentina.

[3]Center of Investigation and Transference of National Northwest University of Buenos Aires (CITNOBA), National Northwest University of Buenos Aires (UNNOBA)-CONICET, Junín, Buenos Aires, Argentina.

[4]Tisioneumonology Division, F.J. Muñiz Hospital, Buenos Aires, Argentina.

*** Correspondence:**
Verónica Edith García
vgarcia@qb.fcen.uba.ar

1 Supplementary Figures and Tables

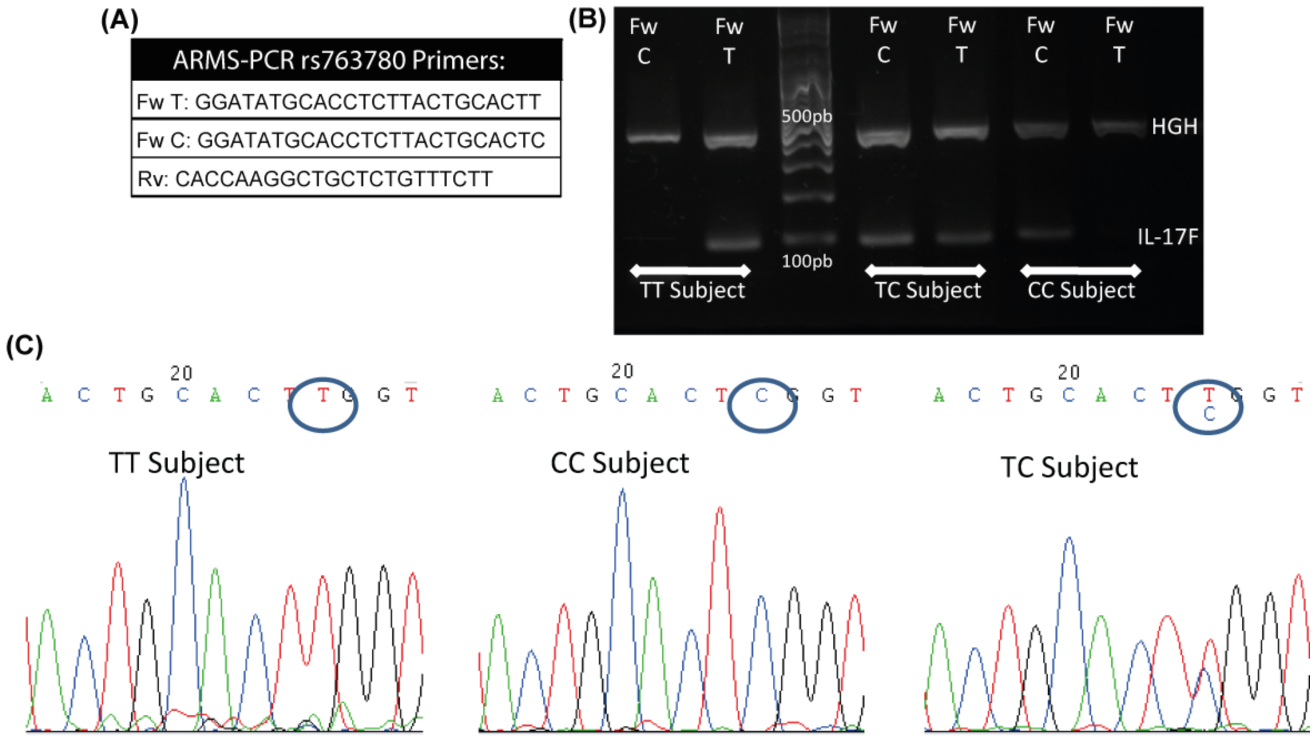

Supplementary Figure S1. *IL17F* rs763780 SNP genotyping by ARMS-PCR method. (A) Primer sequences (two Forward primers and one common Reverse primer) designed to specifically amplify a 106 pb amplicon that discriminates both alleles of the rs763780 SNP. Primer sequences were designed by the BeaconDesigner 7.2 software (Premier Biosoft International). **(B)** Image of an agarose gel displaying the PCR products obtained from three individuals with different genotypes for the SNP under study. PCR positive control: Human Growth Hormone (*HGH*) gene fragment (440 bp). rs763780 genotypes were assessed from the presence/absence of PCR amplicon corresponding to the specific allele (T/C) on 1.5% agarose gel stained with SYBR Green. **(C)** DNA sequencing of the amplicons obtained from three individuals with different genotypes for the SNP. Primers specificity of the rs763780 SNP were confirmed by direct sequencing of the amplified *IL17F* gene fragment by Sanger method (ABI 3130xl GeneticAnalyzer, Applied Biosystems), and a 100% concordance was obtained among the results obtained from ARMS-PCR and DNA sequencing.

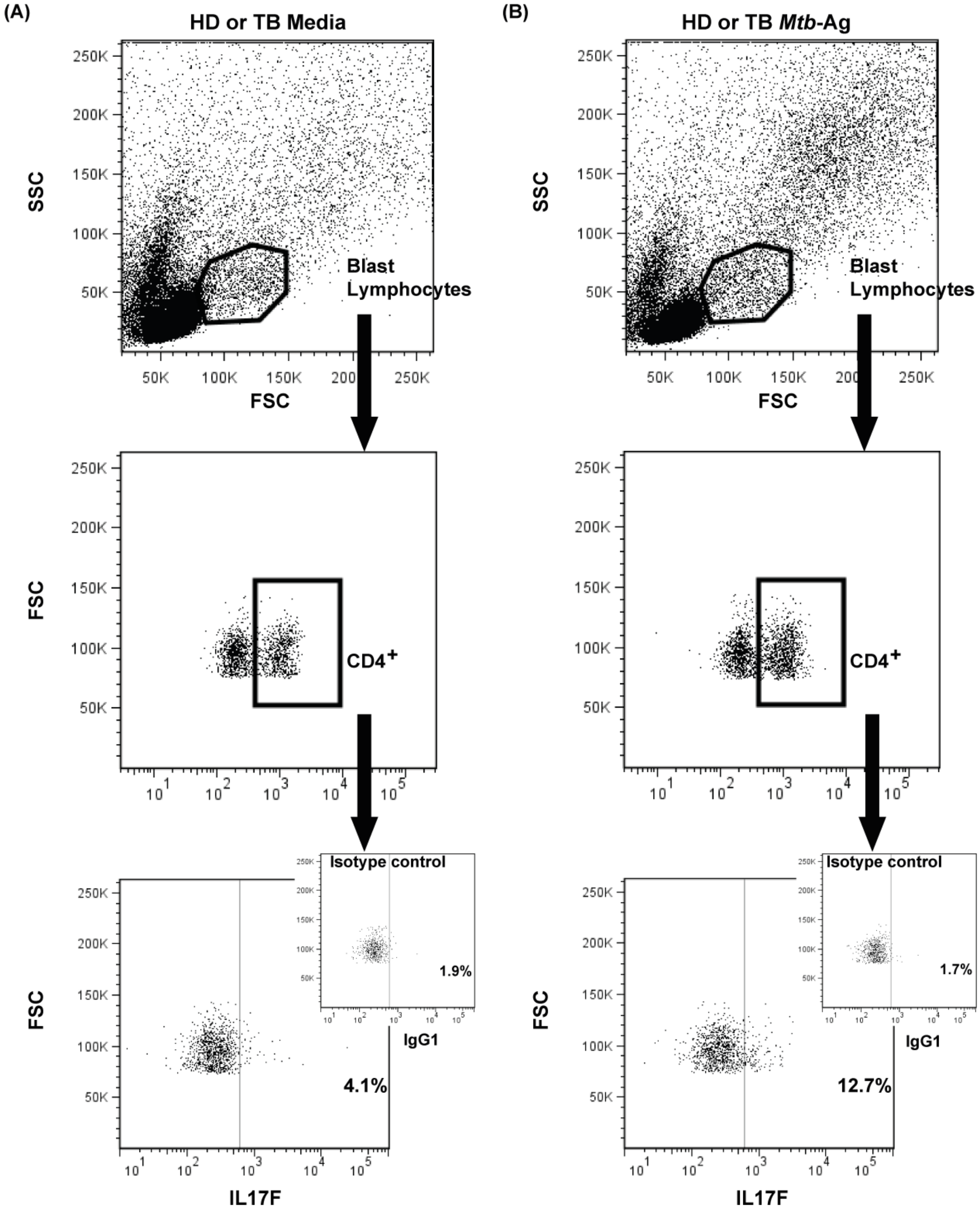

Supplementary Figure S2. Gating strategy used in flow cytometry analysis to detect the production of IL17F after *Mtb*-Ag stimulation. PBMCs were cultured with media **(A)** or with *Mtb*-Ag **(B)**. T cells were identified by first gating on blast lymphocytes based on their forward and side scatter properties (FSC and SSC, respectively). Then, $CD4^+$ cells were discriminated according to FITC fluorescence. Finally $IL17F^+$ cells were determined by staining with specific antibodies bound to eFluor660 fluorophore. Dot plots from a representative healthy donor (HD) are shown. The insets display the isotype.

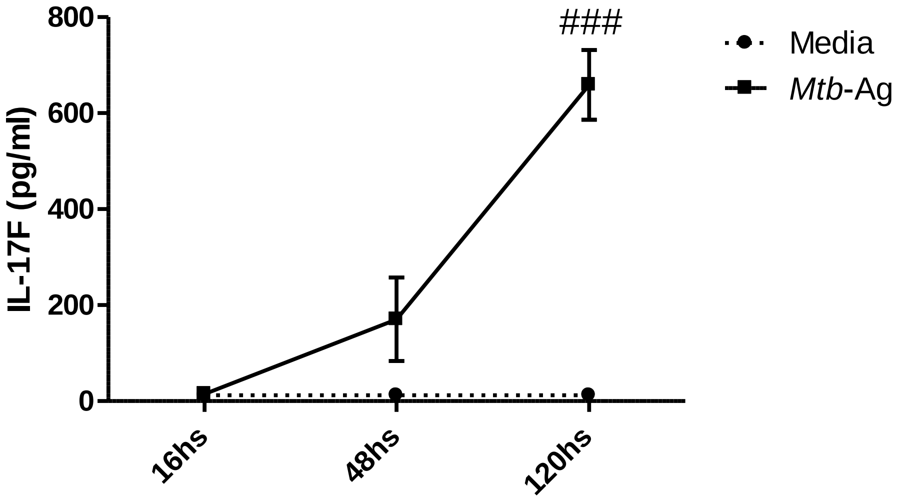

Supplementary Figure S3. IL17F secretion by *Mtb*-Ag stimulated PBMCs at different time points. Peripheral Blood Mononuclear Cells from healthy donors (n=6) were stimulated for 16 h, 48 h and 120 h either alone or with a lysate of *Mtb* (*Mtb*-Ag). Afterwards, IL17F production was determined in culture supernatants by ELISA. Each point represents the Mean ± Standard Error of the Mean (SEM). Statistical differences were calculated using the nonparametric Kruskal-Wallis test for unpaired samples, with the Dunn's Multiple Comparison Post-Test ($^{###}$ p-Value<0.001).

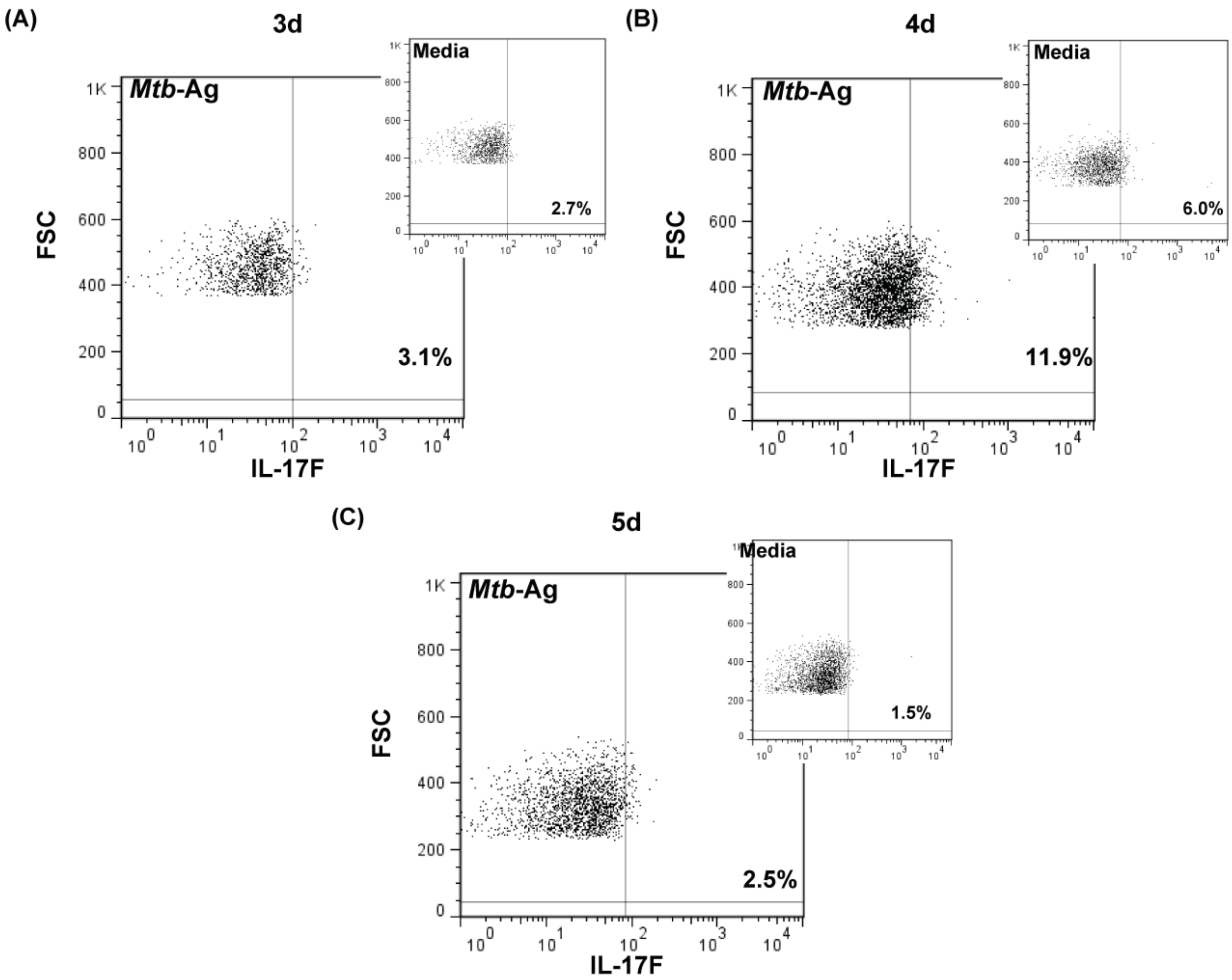

Supplementary Figure S4. IL17F production by PBMCs from a TB after three, four or five days of *Mtb*-Ag stimulation. Peripheral Blood Mononuclear Cells from a TB were cultured for **(A)** three, **(B)** four or **(C)** five days either alone or with a lysate of *Mtb* (*Mtb*-Ag). Afterwards, IL17F production was determined by Flow Cytometry. Representative dot plots are shown. IL17F secretion was determined by first gating on blast lymphocytes by light scatter, and then by gating on $CD4^{+}$ T cells.

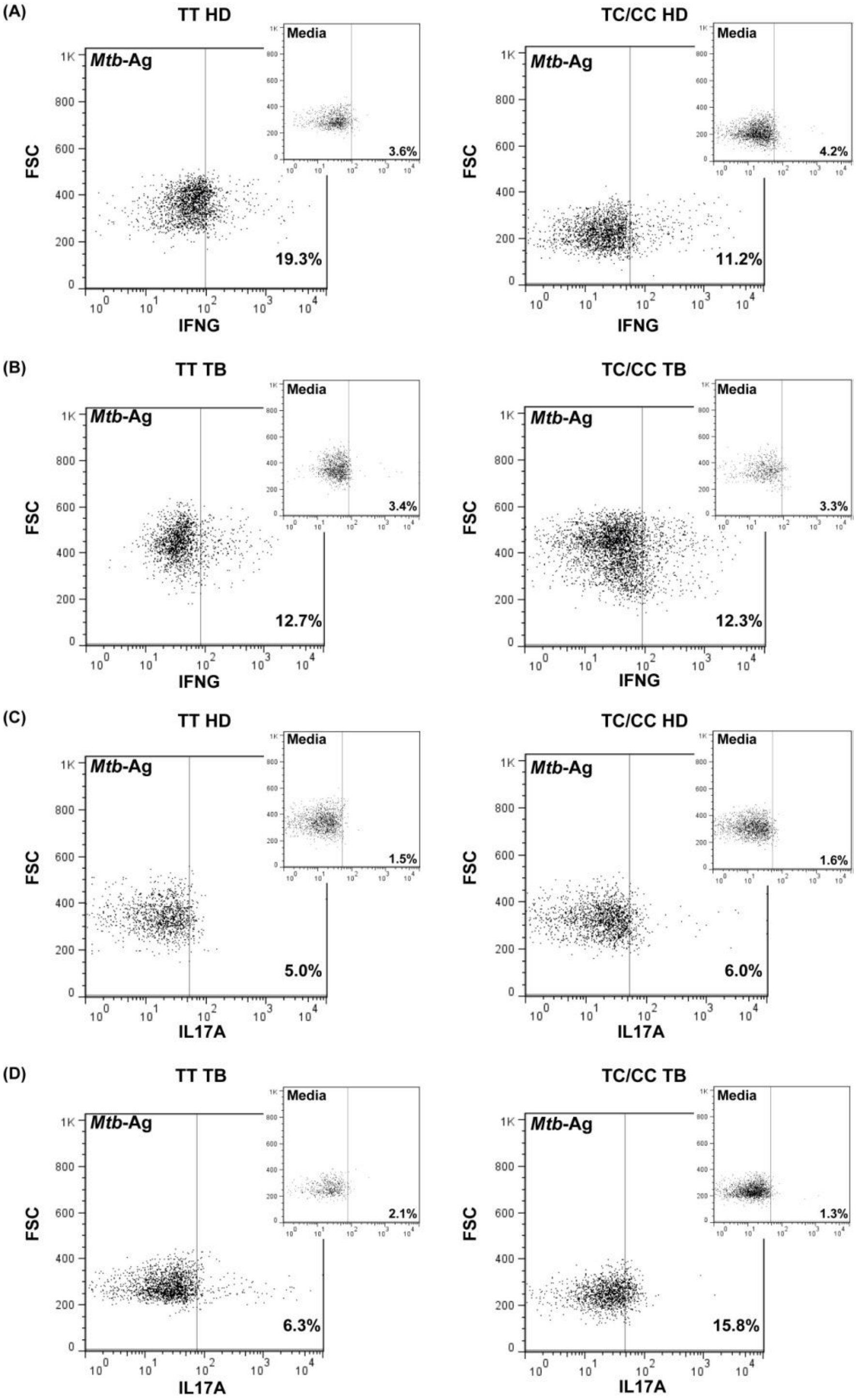

Supplementary Figure S5. IFNG and IL17A production by CD4$^+$ T cells in individuals carrying different genotypic variants of the *IL17F* rs763780 SNP. Peripheral Blood Mononuclear Cells from healthy donors (HD) and tuberculosis patients (TB) carrying the different genotypes of the rs763780 SNP were stimulated for five days with *Mtb*-Ag, and IFNG **(A-B)** and IL17A **(C-D)** secreting T lymphocytes were determined by Flow Cytometry. Representative dot plots for HD **(A and C)** and TB **(B and D)** carrying the different genotypes of the rs763780 SNP are shown. IFNG and IL17A secreting cells were determined by first gating on lymphocytes by light scatter, and then by gating on CD4$^+$ T cells.
